# Chemically synthesized chevron-like graphene nanoribbons for electrochemical sensors development: determination of epinephrine

**DOI:** 10.1038/s41598-020-71554-1

**Published:** 2020-09-03

**Authors:** Raquel Sainz, María del Pozo, Manuel Vilas-Varela, Jesús Castro-Esteban, María Pérez Corral, Luis Vázquez, Elías Blanco, Diego Peña, José A. Martín-Gago, Gary J. Ellis, María Dolores Petit-Domínguez, Carmen Quintana, Elena Casero

**Affiliations:** 1 Departamento de Química Analítica y Análisis Instrumental, Facultad de Ciencias, Campus de Excelencia de la Universidad Autónoma de Madrid, c/ Francisco Tomás y Valiente, Nº7, 28049 Madrid, Spain; 2 Centro Singular de Investigación en Química Biolóxica e Materiais Moleculares (CIQUS) and Departamento de Química Orgánica, Universidade de Santiago de Compostela, 15782 Santiago de Compostela, Spain; 3 ESISNA group, Instituto de Ciencia de Materiales de Madrid (ICMM-CSIC), c/ Sor Juana Inés de la Cruz Nº3, 28049 Madrid, Spain; 4 Departamento de Física de Polímeros, Elastómeros y Aplicaciones Energéticas, Instituto de Ciencia y Tecnología de Polímeros (ICTP-CSIC), c/ Juan de la Cierva, 3, 28006 Madrid, Spain

**Keywords:** Sensors, Electrochemistry, Graphene, Scanning probe microscopy

## Abstract

We employ chevron-like graphene nanoribbons (GNRs) synthesized by a solution-based chemical route to develop a novel electrochemical sensor for determination of the neurotransmitter epinephrine (EPI). The sensor surface, a glassy carbon electrode modified with GNRs, is characterized by atomic force microscopy, scanning electron microscopy and Raman spectroscopy, which show that the electrode surface modification comprises of bi-dimensional multilayer-stacked GNRs that retain their molecular structure. The charge transfer process occurring at the electrode interface is evaluated by electrochemical impedance spectroscopy. The sensor is applied to the determination of EPI, employing as an analytical signal the reduction peak corresponding to the epinephrinechrome–leucoepinephrinechrome transition (E = − 0.25 V) instead of the oxidation peak usually employed in the literature (E =  + 0.6 V) in order to minimize interferences. The results obtained demonstrate that chevron-like nanoribbons synthesized by solution methods exhibit reliable electrocatalytic activity for EPI determination. Using differential pulse voltammetry, we obtain a linear concentration range from 6.4 × 10^–6^ to 1.0 × 10^–4^ M and a detection limit of 2.1 × 10^–6^ M. The applicability of the sensor was evaluated by determining EPI in pharmaceutical samples with satisfactory results.

## Introduction

Due to their outstanding properties, carbon-based nanomaterials, such as graphene and carbon nanotubes, have been widely employed in electrochemical sensor development, promoting continuous advances in this area^1–6^. In contrast, reports concerning the application of graphene nanoribbons (GNRs) to electrochemical sensing are still scarce, as can be seen in a review devoted to this issue, published in 2018, where only around 40 references are included describing several GNR-based (bio)sensors^7^.

GNRs consist of narrow strips of *sp*^2^ bonded carbon atoms, with band gaps depending on the GNRs width and their edge structure. An important challenge for the different fabrication methods is to achieve both a reliable control over the final width and edge structure of the resulting GNR, along with a workable large-scale production method. The synthesis of GNRs can be performed by top-down and bottom-up approaches^8–16^. The top-down methodology consists of obtaining GNRs with specific size and shape from precursors such as graphene sheets or carbon nanotubes. Employing this approach, GNRs can be obtained by unzipping carbon nanotubes, by cutting graphene with catalytic particles or by lithographic patterning methods. The bottom-up approach leads to GNRs formation by assembling molecular building blocks. The assembly can be achieved by on-surface synthesis or solution-based organic chemistry. Comparing both methodologies, the bottom-up approach leads to more structurally well-defined and homogenous GNRs than top-down methodologies, which lack structural precision and are also limited by low yields^1^.

The semiconducting behavior with a tunable bandgap and the excellent transport properties make GNRs obtained by bottom-up methodologies suitable candidates for applications related to the fabrication of nanoscale electronic devices. GNRs have also attracted considerable attention in other research fields since, compared with carbon nanotubes, they present a higher area-normalized edge-plane and sometimes a great number of rich edge defects and chemically active sites, which make them a good candidate for electroanalytical applications. As mentioned above, there are few reports on the development of GNR-based electrochemical sensors for the determination of compounds of interest, such as ampyra, diazonin, nimesulide, 3,4-dihydroxyphenylacetic acid, baicalein, levodopa, glucose and ethanol^17–24^.

Epinephrine (EPI), also known as adrenaline, is one of the three catecholamine species that are known: EPI, norepinephrine and dopamine. EPI is a neurotransmitter secreted by the adrenal cortex in the adrenal gland. It acts via adrenergic receptors and performs an important role stimulating a range of actions of the sympathetic nervous system (SNS) such as psychomotor activity or emotional processes. A wide variety of studies reveal that variations of EPI levels in nervous tissues or biologic fluids are diagnostic symptoms of several diseases^25–28^. On the other hand, EPI is commercialized as a pharmaceutical product employed in life-threatening emergencies such as severe reactions to allergens and even anaphylactic shocks. Therefore, the development of quantitative methods to determine EPI in biologic and pharmaceutical samples is of significant interest. In this respect, different analytical procedures have been developed for extraction and determination of catecholamines and their metabolites. Among them, capillary electrophoresis, electrochemical, optical and chromatographic techniques are usually employed^29–32^.

Remarkably, in all previous reports concerning development of GNR-based electrochemical sensors for determination of compounds of interest, specifically over the last 2 years, GNRs have been obtained by longitudinal unzipping of carbon nanotubes. Moreover, most of the sensors include in their design, in addition to GNRs, other nanomaterials such as graphene or metallic nanoparticles, which lead to complicated systems. In this sense, in the present work our aim is to evaluate the applicability of well-defined GNRs synthesized by a bottom-up approach consisting in a solution-based methodology, without any combination with other nanomaterials, in an easily scalable electrochemical sensor development for epinephrine determination.

## Results and discussion

### Morphological and structural characterization of the GNR-modified electrode

**Figure 1:**
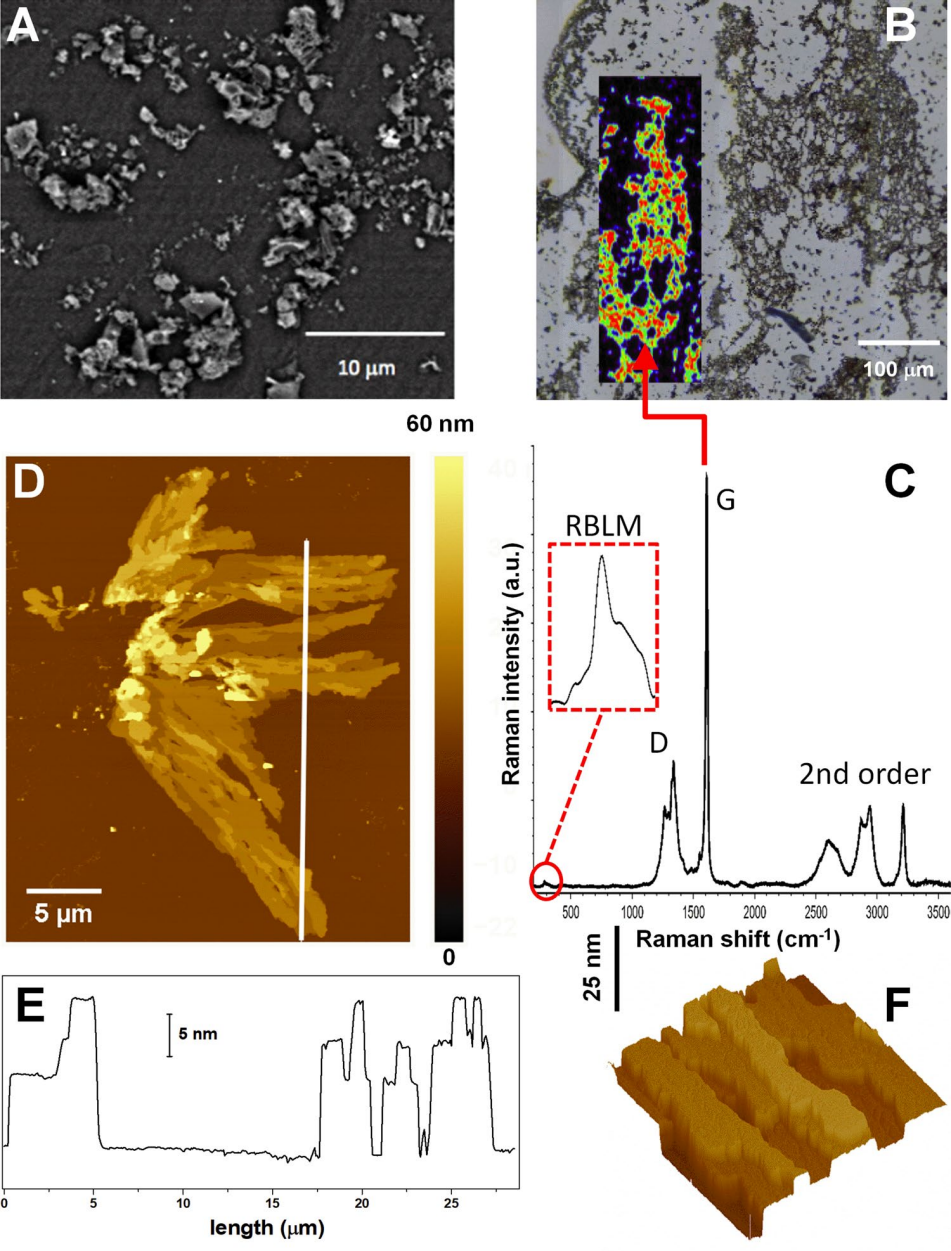
(**A**) SEM and (**B**) optical images of as-deposited GNRs on glassy carbon and gold surfaces, respectively. The inset in (**B**) is a false-colour map generated from the integrated intensity of the Raman G mode (marked in **C**) at 1,610 cm^−1^, superimposed over the optical image, showing the network-like distribution of the nanoribbons. (**C**) A typical Raman spectrum recorded from the GNR deposit (in **B**). In the inset, the RBLM mode at around 284 cm^−1^ is highlighted. (**D**) 27.5 × 35 μm^2^ AFM image taken on a small GNR aggregate deposited on the silicon surface. (**E**) Surface profile taken along the solid line depicted in (**D**). The left of the profile corresponds to the bottom extreme of the line. (**F**) 3 × 3 μm^2^ AFM image shown in 3D representation and with illumination to highlight the multilayer morphology.

The GNR-modified sensor electrodes were prepared as described in detail in “Preparation of graphene nanoribbons modified glassy carbon electrode” section of the “Materials and methods” section. Morphological characterization was carried out at different scales of the GNR flakes using SEM and AFM, complemented by a spectroscopic study to evidence their molecular nature. Figure 1A shows a SEM image of the GNR deposited on glassy carbon electrodes (GCE). The image shows that the GNRs group together in micro and sub-micrometer aggregates dispersed on the electrode surface. Interestingly, some of them show a very smooth surface. To study their molecular nature, Raman spectra were recorded from GNRs deposited on a gold substrate (see “Materials and methods” section). This surface was chosen to avoid spectral contributions from the glassy carbon substrate. The optical image in Fig. 1B shows that the aggregates cover the surface with a morphology that consists of interdigitated structures, forming a kind of network, as it has been previously reported^33^. Raman spectra were recorded from different zones of this network, and a representative spectrum is given in Fig. 1C. The inset in Fig. 1B represents a false-colour map generated from the integrated intensity of the Raman G mode (marked) that appears at 1,610 cm^−1^ and highlights the micron-scale network-like distribution of the nanoribbons. As well as the G-mode, several other Raman bands characteristic of GNRs can be clearly observed in the spectrum in Fig. 1C^34–37^. The defect mode region is structured with a CH-edge bending at 1,266 cm^−1^ and D-mode at 1,340 cm^−1^, and a combination of higher order modes can be identified that is unique to GNRs^34^, corresponding to the overtone and combination bands, 2D and D + D at 2,605 and 2,685 cm^−1^, the D + G modes at 2,875 and 2,942 cm^−1^ and a 2G mode at 3,216 cm^−1^. Finally, at low frequency a radial breathing-like mode (RBLM) is observed and highlighted in the spectrum at around 284 cm^−1^, a frequency that corresponds to ribbons with a width in the order of 10–12 Å according to continuum rod models^34–36,38^.

AFM was used to obtain more structural details about the smallest flakes that cannot be distinguished by the SEM and optical micrographs. For that purpose, we employed silicon as substrate (see “Materials and methods” section). Figure 1D shows a small and relatively flat aggregate of GNRs deposited on a silicon surface. It extends up to 34 μm in the vertical y-axis and almost 20 μm along the horizontal x-axis. It is formed by a stacking of elongated flat flakes in a multilayer-like arrangement, with thickness (AFM apparent height) ranging from 8 to 34 nm. The multilayer arrangement is better observed in the surface profile in Fig. 1E, taken along the solid line in Fig. 1D, showing a flat surface. This can also be appreciated in Fig. 1F where the different height levels of the stacked arrangement are better shown. When the surface roughness is measured on the top terraces of the flakes, the value obtained is < 0.4 nm confirming the extreme flatness of the flakes. The structural values derived from our AFM analysis are consistent with π−π stacked nanocrystallites of GNRs^39^.

**Figure 2:**
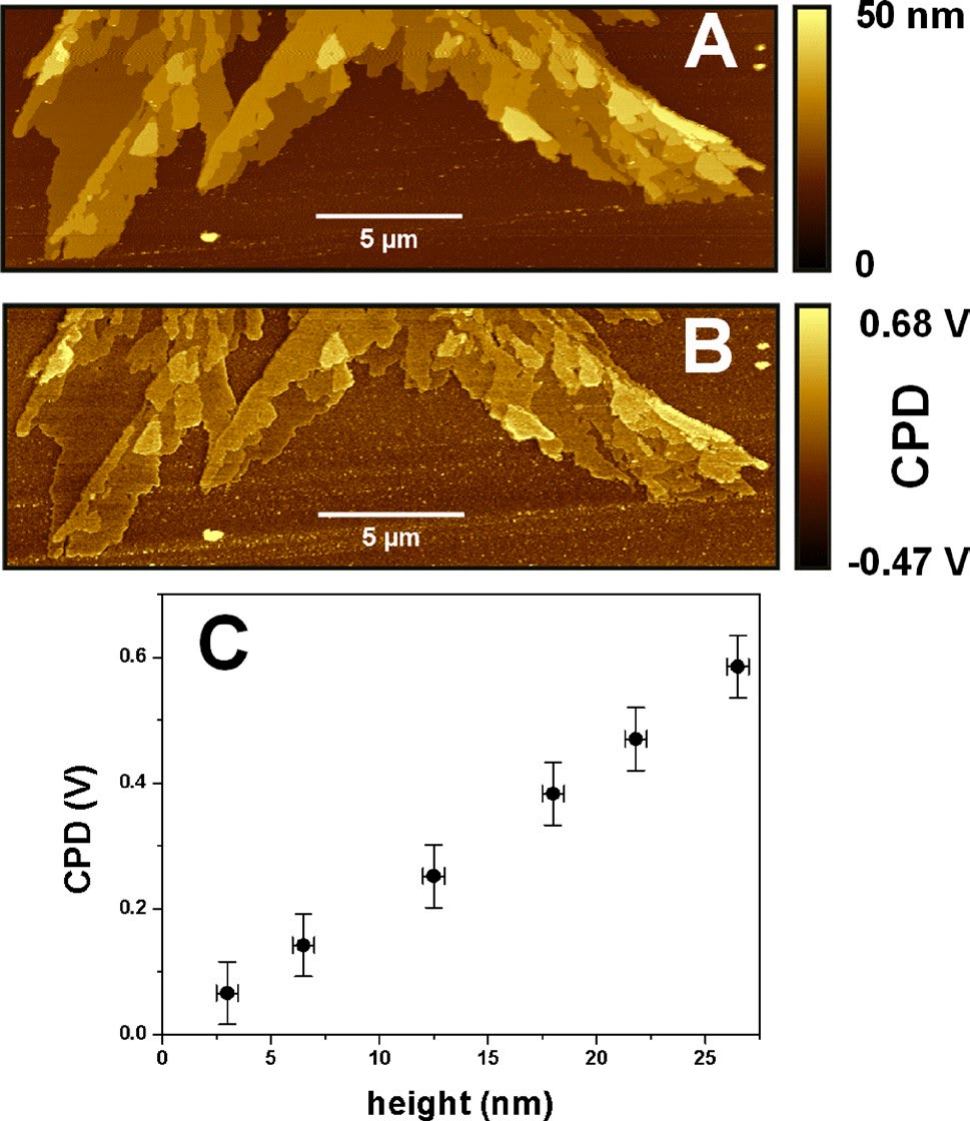
27 × 9 μm^2^ (**A**) AFM and (**B**) KFM taken simultaneously. Note how the CPD signal increases with the multilayer thickness. (**C**) Plot of the average CPD value versus the height (i.e. thickness) range.

In a further step in the AFM characterization, we have focused our attention on a stacked flake to characterize its electronic properties by KFM. Figure 2A,B show the simultaneous AFM and KFM images recorded on this multilayer morphology. It is evident that there is a correlation between the heights of the top terrace (i.e. of the thicknesses of the multilayer) and the corresponding contact potential difference (CPD) shown in the KFM image.

From these images a quantitative analysis performed with the Gwyddion software^40^ allows us to obtain the change in the CPD signal from the SiO_2_ surface to the GNR flakes due to their different thickness values (Fig. 2C). A linear dependence of the CPD with the thickness is observed up to 27 nm, with a notable homogeneity of the CPD signal over each terrace. CPD usually represents the work function difference between both materials, which is modified due to the charge transfer between the substrate and the flake. A linear dependence of the CPD with thickness in a 2D system has been reported at the interface in the case of many different flakes, reaching saturation to the bulk value after about 10–20 multilayers^41–44^. However, Fig. 2C shows that here the linear behavior extends to more than 30 nm. We believe this unusual trend could be due to the semiconducting nature of the GNR, with an approximate thickness-independent band-gap of 3.6 eV^45,46^.

### Electrochemical characterization of the GNR-modified electrode

**Figure 3:**
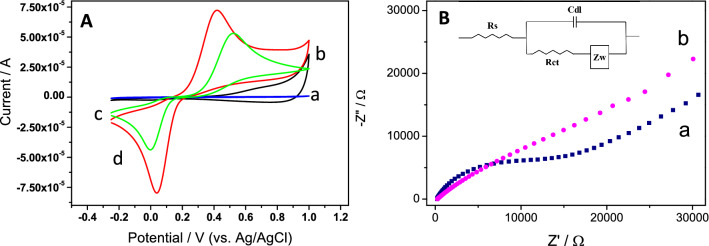
(**A**) Cyclic voltammograms in a 0.1 M phosphate buffer solution at pH 7.0 of a bare and a GNR-modified GCE in the absence (a and b, respectively) and in the presence (c and d, respectively) of 2.5 mM HQ. Scan rate 0.1 V s^−1^. (**B**) Nyquist plots of (a) bare GCE and (b) GNR-modified GCE in 0.1 M phosphate buffer (pH 7) containing 2.5 mM HQ. Amplitude ± 10 mV. Frequency range: 10^5^–10^–2^ Hz. Applied potential: 0.56 V. Inset: Randles electrical equivalent circuit where Rs (electrolyte resistance), R_CT_ (charge transfer resistance), C_dl_ (double layer capacitance) and Z_W_ (Warburg impedance).

In order to evaluate the effect of the as-synthesized GNRs in electrochemical sensing, hydroquinone (HQ) was selected as a typical redox probe. Figure 3A shows the cyclic voltammetric (CV) response of both a bare and a GNR-modified GCE in a 0.1 M phosphate buffer at pH 7.0 in the absence (curves a and b) and in the presence (curves c and d) of 2.5 mM HQ. As can be observed, GCE modification with GNRs (curve b) involves an increase of the capacitive current with respect to the bare GCE (curve a), suggesting that GNRs have been successfully incorporated onto the electrode surface. As one would expect, the cyclic voltammograms obtained for HQ, at both bare and GNR-modified electrodes (curves c and d), exhibit a redox couple corresponding to the hydroquinone/quinone process. Compared with the bare GCE, the anodic and cathodic currents of the GNR-modified electrode are increased by factors of about 1.3 and 2, respectively. Moreover, the anodic and cathodic peak separation of HQ is diminished by 142 mV at GNRs/GCE in comparison with that obtained at GCE. These results indicate that the presence of GNRs on the electrode surface generates an improved response, likely due to an increase of both the charge electron transfer and the effective surface area of the electrode. In order to confirm both points, the interfacial electrochemical properties and the electroactive surface of the bare and the GNR-modified electrode were compared.

The interfacial electrochemical properties were evaluated by electrochemical impedance spectroscopy (EIS) in 0.1 M phosphate buffer (pH 7.0) containing 2.5 mM HQ. The Nyquist plot of the bare electrode (Fig. 3B, a) consists of a semicircle and a linear part. The semicircle is ascribed to the electron transfer-limited process and its diameter is related to the electron transfer resistance of the electrode interface. From the fit of these data, considering the Randles electrical equivalent circuit shown in the inset of Fig. 3B, a R_CT_ value of 14,828 Ω was obtained. The linear part of the plot, obtained at low frequencies, corresponds to the diffusion process. For the GNR-modified electrode, a simple straight line dependence is obtained (Fig. 3B, b), showing that the charge transfer is clearly improved since a diffusion-limited transport process is observed.

On the other hand, the electrochemically active surface was evaluated employing an outer sphere redox probe, Ru(NH_3_)_6_^2+/3+^, since for this system the electron transfer does not depend on an interaction with a functional group or surface site^47^. Cyclic voltammograms registered at different scan rates for GC and GNRs/GC electrodes in a 1 M KCl solution containing 1 mM Ru(NH_3_)_6_^2+/3+^ were obtained. An example of CV at 10 mV/s is displayed in Supplementary Fig. S1. The measured peak current is related with the electrochemical surface area through the Randles–Sevick equation^48,49^

$$ {\text{Ip}} = \left( {2.69 \times 10^{5} } \right)\;{\text{n}}^{3/2} \;{\text{A}}\;{\text{D}}^{\raise.5ex\hbox{$\scriptstyle 1$}\kern-.1em/ \kern-.15em\lower.25ex\hbox{$\scriptstyle 2$} } \;{\text{v}}^{\raise.5ex\hbox{$\scriptstyle 1$}\kern-.1em/ \kern-.15em\lower.25ex\hbox{$\scriptstyle 2$} } \;{\text{C}} $$

where Ip is the measured peak current, n the number of electrons, A the electrochemical surface area, D the diffusion coefficient of Ru(NH_3_)_6_^2+/3+^ (7.9 × 10^–6^ cm^2^ s^−1^ at 22 °C^50^), v the scan rate, and C the concentration of the redox compound. From the slope of the corresponding I_p_ versus v^1/2^ plots (Supplementary Fig. S1), electrochemical surface areas of (0.062 ± 0.002) cm^2^ and (0.089 ± 0.006) cm^2^ were obtained for the bare and the modified GC electrodes, respectively. These values show that an increase, close to 44%, of the effective area is produced as a result of the GNRs presence on the electrode surface.

### Electrochemical behavior of epinephrine on GC and GNRs/GC electrodes

**Figure 4:**
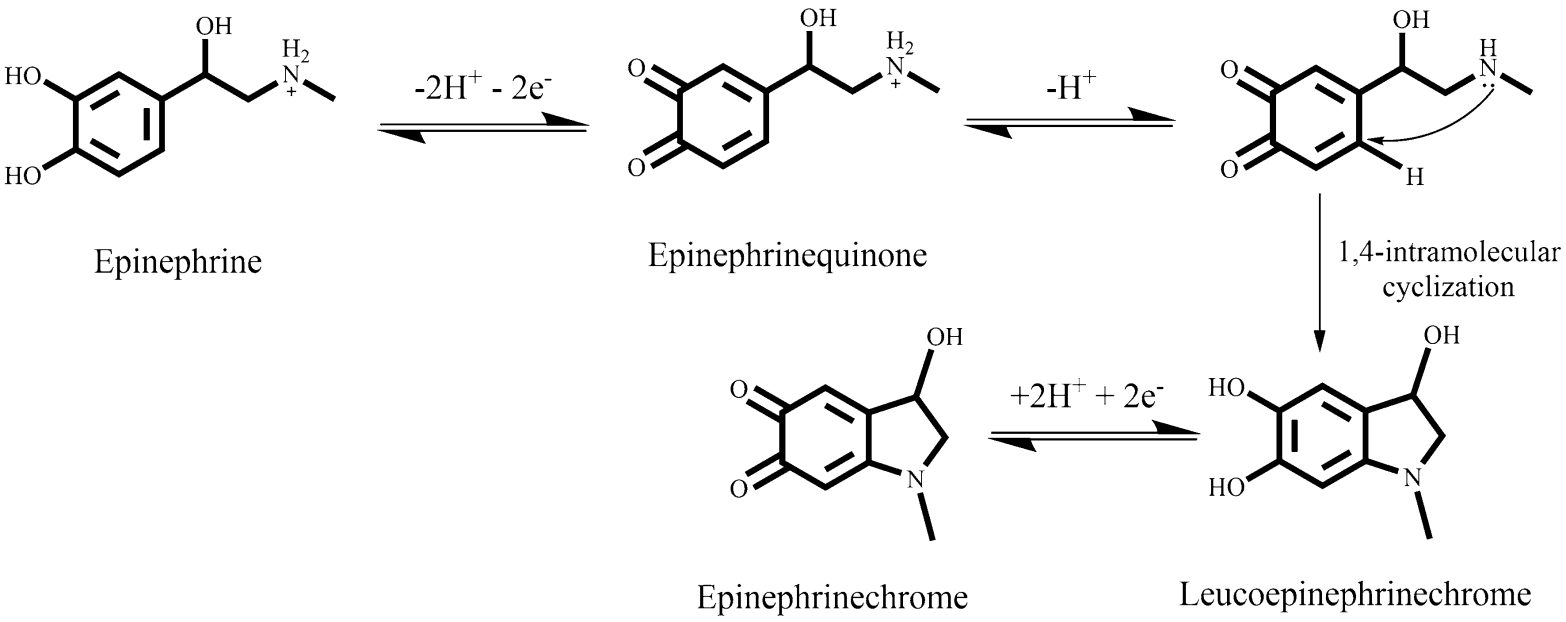
Epinephrine oxidation according to ECE (Electron transfer—Chemical reaction—Electron transfer) model. The chemical structures were drawn using ChemDraw v16, https://www.perkinelmer.com/.

As shown in Fig. 4, EPI is able to undergo oxidation reactions, where ortho-quinones and cyclization products are involved^51^.

**Figure 5:**
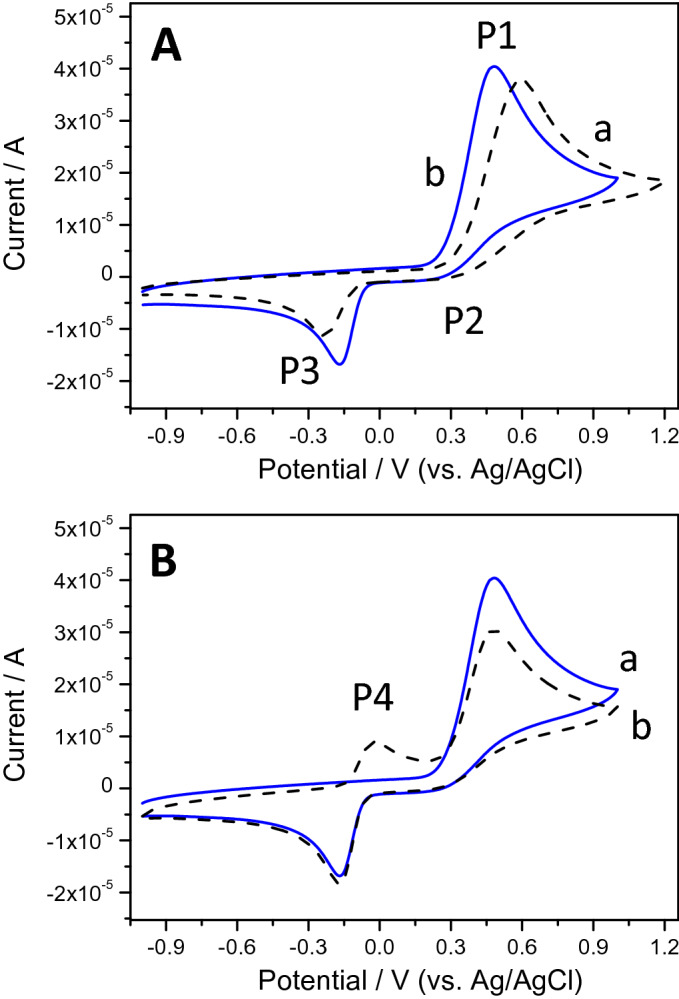
(**A**) Cyclic voltammograms of (a) a bare GCE and (b) a GNR-modified GCE in a 0.1 M phosphate buffer at pH 7.0 containing 1 mM EPI. (**B**) First (a) and second (b) scans of a GNR-modified GCE in the same solution. Scan rate 0.1 V s^−1^.

Figure 5A shows the response of a bare GCE (curve a) and modified with GNRs (curve b), in a 0.1 M phosphate buffer solution containing 1 mM EPI. For both cases, a peak (P1), corresponding to EPI oxidation is observed in the anodic scan. As it is shown in Fig. 4, the epinephrinequinone resulting from EPI oxidation can remain as such or can undergo a nucleophilic attack of the nitrogen atom (Michael addition) with an irreversible 1,4 intramolecular cyclization, leading to the formation of an unstable species, leucoepinephrinechrome, which is easily oxidized to epinephrinechrome^52,53^.

As a result, in the reverse scan, two different cathodic peaks could appear: a first (P2) at around + 0.3 V, due to the reduction of the epinephrinequinone, which has not suffered the cyclization process to EPI, and a second (P3), ascribed to the reduction of previously formed epinephrinechrome. The rate and extent of the Michael addition are highly dependent on the experimental conditions, mainly on the pH value, as will be discussed later. Under the experimental conditions employed for recording the CV displayed in Fig. 5, the Michael addition is highly promoted, therefore, the current intensity of P3 is much higher than that of P2, which is barely appreciable. Regarding the effect of GNRs, their presence on the GCE induces an increase in the current intensity of the P1 and P3 peaks as well as a shift in their peak potential values (130 mV and 80 mV for P1 and P3, respectively), leading to an improvement in the reversibility of the system. In Fig. 5B, the first (curve a) and second scan (curve b) obtained with the GNRs/GCE system are compared. As a counterpart of P3, in the second scan a new oxidation peak (P4) appears, corresponding to the oxidation of leucoepinephrinechrome. These results are in concordance with an ECE (Electron transfer—Chemical reaction—Electron transfer) mechanism. Considering these results, EPI determination can be carried out employing the current intensity measured for P1, P3 or P4 peaks. As it will be explained later, the P3 current intensity is selected as analytical signal.

The effect of scan rate on the EPI electrochemical response at GNRs/GCE was investigated in the range comprised between 10 and 300 mV s^−1^. The resulting CVs, recorded for 1 mM EPI in phosphate buffer, are shown in Supplementary Fig. S2. The current intensity of peaks P1, P3 and P4 changes linearly with the square root of the scan rate (v^1/2^), indicating that the electrochemical transitions on the modified electrode surface are mainly controlled by diffusion processes. In the inset of Supplementary Fig. S2, the current intensity of P3 versus v^1/2^ is plotted, showing a linear relationship through the following equation, I_P3_ = −2.75 v^1/2^ + 5.47 (R^2^ = 0.998).

### Analytical methodology optimization for EPI determination on GNRs/GCE sensor

EPI determination has been usually performed employing EPI oxidation current (peak P1), which, in our case, appears at + 0.6 V. In order to avoid this high overpotential, we focus on employing the reduction peak corresponding to epinephrinechrome–leucoepinephrinechrome transition (P3) as analytical signal.

#### pH optimization

The optimization of the pH value becomes of critical importance since the nucleophilic character of the EPI amine group, which is necessary for Michael addition, depends on its deprotonation. Therefore, depending on pH, different values for the current intensity and the potential value of the reduction peak corresponding to epinephrinechrome–leucoepinephrinechrome transition (peak P3) are expected.

For pH optimization, seven buffer solutions, containing 1.0 mM EPI, were prepared in the pH range of 3.0–9.0 and employed for recording the electrochemical response, but for clarity sake, only three of them (pH 3, 6, 8) are displayed in Supplementary Fig. S3. As can be observed, the potential values of all the peaks shifted towards negative E values as pH increased, according to the Nernst equation. The corresponding equation of the fitting of E_P3_ vs pH (E_P3_ = 0.41–0.074 pH, r = 0.994) indicates that the same number of protons and electrons are involved in the process, according to the mechanism shown in Fig. 4.

On the other hand, when the pH is highly acidic, P3 has almost disappeared and the current intensity of P2 is maximum since the epinephrinequinone is largely protonated, precluding the cyclization reaction. When the pH increases (pH 6.0), deprotonation takes place leading to an increase of the P3 current intensity and a decrease of the P2 intensity due to the formation of the cyclized product. Above this pH value, the current intensity of the epinephrinechrome–leucoepinephrinechrome couple (P3, P4) increases, reaching a maximum at pH 8.0. Thus, for EPI determination, a pH value of 8.0 was selected.

#### Instrumental parameters optimization

In order to determine the concentration of EPI, we employ the differential pulse voltammetry (DPV) technique due to its higher sensitivity compared to cyclic voltammetry. Since we focus on the reduction peak corresponding to epinephrinechrome-leucoepinephrinechrome transition (peak P3), prior to the measurement we need to form epinephrinechrome from EPI. Therefore, our analytical method implies a first step, consisting in the application of an oxidation potential (E_0_) during a fixed time (t). In order to optimize these parameters, several potential and time values were assayed and the corresponding P3 current intensity was measured, obtaining a maximum value for an initial applied voltage of 0.6 V during 60 s prior to measurement. The rest of DPV instrumental parameters, initial potential (E_i_), final potential (E_f_), amplitude (a) and scan rate (v) were optimized in a similar way in order to obtain well-defined peaks (see Supplementary Table S1).

### Influence of EPI concentration in the peak currents: analytical parameters

**Figure 6:**
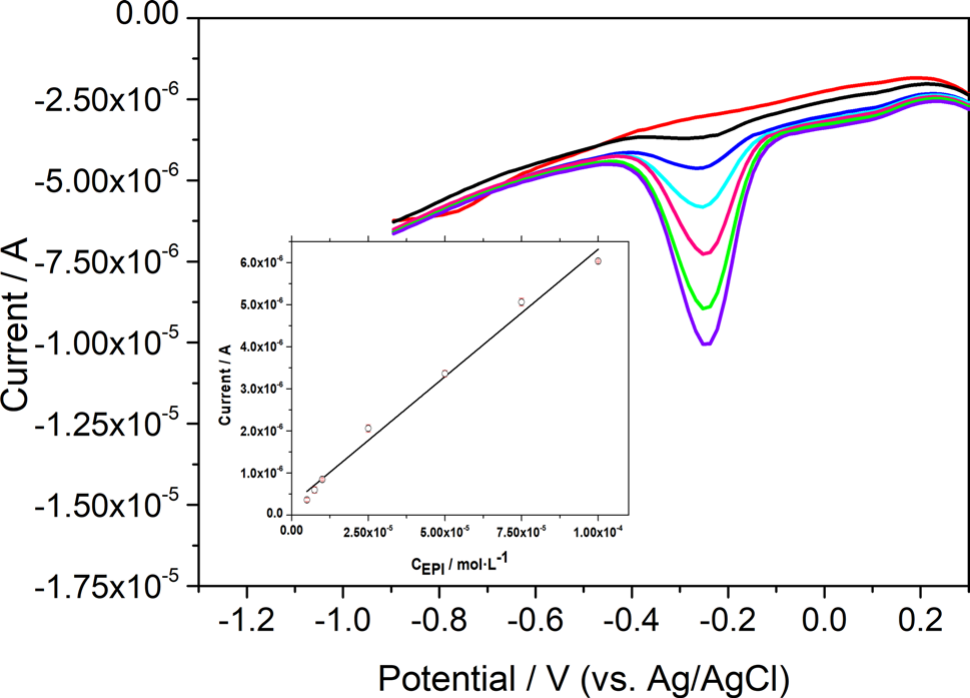
DPV response of GNRs/GCE in 0.1 M phosphate buffer solution containing different concentration of EPI. Inset: calibration plot.

Under optimized conditions, we measured the electrochemical response of the GNRs/GCE sensor towards increasing concentrations of EPI (Fig. 6). As can be observed in the inset of Fig. 6 that displays the data of current intensity vs. EPI concentration, we obtained a linear relationship over the concentration range of 6.4–100 µM whose equation was I (A) = 2.62 × 10^–7^ + 0.0605 C_EPI_ (M) (r = 0.990). The detection (LOD) and quantification limits (LOQ) values for the DPV detection of EPI, obtained as the ratio between three and ten times the standard deviation of the blank signal and the sensitivity, were found to be 2.1 µM and 6.4 µM, respectively. In Supplementary Table S2, the LOD and linearity range of several electrochemical methods found in the literature for EPI determination are compared with our method^54–67^. As it can be observed, the developed sensor shows good analytical properties similar to those obtained for others also based on nanomaterials. Moreover, it is worth noting that, on the one hand, our sensor design is very simple since it only requires the modification of the electrode with the nanoribbons, while most of the sensors displayed in the table require the use of a combination of different nanomaterials, in more complicated designs. On the other hand, we have carried out EPI determination employing as electrochemical signal the current intensity of the cathodic peak P3 instead of the anodic peak, which could be more adequate to avoid interferences.

Finally, accuracy and precision (repeatability and reproducibility) of the sensor were evaluated at different levels of EPI concentrations in the linear range. Concerning the repeatability, DPV measurements were recorded with the same modified electrode. Relative standard deviation (R.S.D.) values minor or equal to 5.2% (n = 20) were obtained, indicating that GNRs/GCE exhibits a notable repeatability. Additionally, to determine the reproducibility, DPV measurements were recorded employing different GNRs/GC electrodes, obtaining R.S.D. values (n = 5) lower than 6.9%. Relative errors minor than 13% were obtained, in all cases assayed. Finally, storage stability was also evaluated by recording the sensor response 20 days after preparation, displaying 90% of the original response. The sensor was kept at 4 °C after preparation.

### Interferences study

In order to evaluate the applicability of the sensor, tartaric acid, sodium metabisulphite and sodium chloride were studied as interferents since they are present in the pharmaceutical real samples. In addition, we also studied dopamine, since this molecule presents a structure analogous to EPI. We recorded the GNRs/GCE response before and after adding increasing amounts of each compound to a solution containing 2.5 × 10^–5^ M EPI (see Supplementary Fig. S4). It was considered that a given compound interferes at a concentration level enough to produce a variation on the initial analytical signal higher than the maximum relative error (Er = 13%, see red lines in Supplementary Fig. S4). Accordingly, we found that the presence of tartaric acid, Na_2_S_2_O_5_, NaCl and dopamine interferes for concentrations higher than 1.2 × 10^–4^ M, 1.2 × 10^–5^ M, 2.4 × 10^–4^ M and 6.3 × 10^–6^ M, respectively.

### Application to real samples: determination of EPI in pharmaceutical product

The applicability of the sensor was tested by measuring the EPI content of a pharmaceutical product. Different volumes of a standard solution of 0.01 M EPI were added to 10.00 mL volumetric flasks containing 0.06 mL of the pharmaceutical sample and made up to the final volume with 0.1 M phosphate buffer (pH 8). DPV measurements, recorded with the GNRs/GCE sensor in the different solutions, are displayed in Supplementary Fig. S5. As can be observed, the current measured at -0.25 V increases with EPI addition according to I (A) = 1.50 × 10^–6^ + 0.0451 [EPI] (M), r = 0.9995 (inset of Supplementary Fig. S5). Comparison between slopes obtained by the standard addition (0.0451 A × M^−1^) and the external calibration (0.0605 A × M^−1^) methods suggests that matrix interference is produced in these conditions. Therefore, EPI determination cannot be performed by direct interpolation in the external calibration plot, but by employing the standard addition. Recoveries obtained are summarized in Supplementary Table S3. The sample was analysed in triplicate obtaining 1.04 (± 0.03) mg mL^−1^ of EPI in the sample, which agrees well with the declared content, demonstrating that the GNRs/GCE sensor can be satisfactory applied to EPI determination in pharmaceutical samples with no sample treatment except dilution.

## Materials and methods

### Chemicals and reagents

All reagents were used without further purification. Epinephrine (EPI), dopamine, hydroquinone (HQ), hexaamin ruthenium (III), tartaric acid, sodium metabisulphite, potassium chloride, sodium chloride and *N*-methyl pyrrolidone (NMP) were purchased from Sigma-Aldrich. As supporting electrolyte, 0.1 M buffer solutions were prepared from ortho-phosphoric or acetic acid in the pH range of 3.0–9.0. For the solution-based synthesis of GNRs, phenanthrene-9,10-quinone, 1,3-diphenylacetone, *N*-bromosuccinimide (NBS), diphenylacetylene, bis(1,5-cyclooctadiene)nickel(0) (Ni (COD)_2_) and iron trichloride were purchased from Sigma-Aldrich.

### Apparatus

Electrochemical measurements, including cyclic voltammetry (CV) and differential pulse voltammetry (DPV), were carried out using a µ-Autolab (type III with GPES software) potentiostat (Utrecht, The Netherlands). EIS measurements were performed using an Eco-Chemie Autolab PGSTAT 302N system with FRA software (Utrecht, The Netherlands). Electrochemical system consisted on a conventional three-electrode cell with an Ag/AgCl (1.0 M KCl) as a reference electrode, a platinum wire as a counter electrode and a GNR-modified glassy carbon electrode (GCE) as working electrode.

Atomic force microscopy (AFM) studies were carried out using two different systems, a Nanocope IIIa (Veeco) equipment and a Nano-Observer (CSInstruments). The former was employed for topographical analysis in which silicon cantilevers (Bruker) with nominal force constant of 40 N m^−1^ and radius of curvature of 8 nm were used. The images were taken in the dynamic mode. The latter equipment was applied to analyze the samples by Kelvin Force Microscopy (KFM). For that purpose Pt-coated cantilevers (ANSCM-PT from AppNano) with a nominal constant force of 1.5 N m^−1^ and a radius smaller than 30 nm, were used. The images taken with the Veeco equipment had 512 × 512 pixels whereas those obtained with Nano-observer had 1,024 × 1,024 pixels. In the KFM mode, it is measured the contact potential difference (CPD) that is related to the changes on the local work function at the surface.

Scanning electron microscopy (SEM) images were obtained with a NOVA NANOSEM 230 equipment (FEI) operating with a low-voltage and high contrast detector (vCD). The landing electron beam energy was 6–7 keV.

Raman spectra were recorded using an *in-*Via Reflex Raman Microscope (Renishaw plc, Wooton-under-Edge, UK) through a 100 × (NA = 0.85) objective lens using a 514.5 nm (2.40 eV) wavelength Ar^+^ laser source with a power of < 2 mW at the sample. Streamline mapping was made at a spatial contrast of approximately the wavelength of the source, and spectral data was analysed using the Renishaw Wire 5.0 software.

Bandelin sonoplus ultrasonic probe was used for homogenizing the nanoribbons suspension.

### Procedures

#### Synthesis of graphene nanoribbons

**Figure 7:**
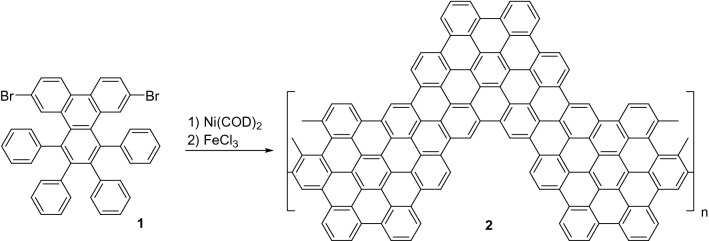
Solution synthesis of chevron-like GNRs. The chemical structures were drawn using ChemDraw v16, https://www.perkinelmer.com/.

For the synthesis of chevron-like GNRs we followed a previously reported procedure^14^. Monomer **1** was obtained from phenanthrene-9,10-quinone in three steps, including bromination with NBS, condensation with 1,3-diphenylacetone, and reaction with diphenylacetylene^14^. Then, Ni(COD)_2_-promoted polymerization of compound **1** followed by FeCl_3_-promoted cyclodehydrogenation led to chevron-like GNRs **2** (Fig. 7).

#### Preparation of graphene nanoribbons modified glassy carbon electrode

Prior to the modification, GCE surface was polished with alumina powder (0.3 µm), sonicated in water/ethanol for 3 min and finally dried with N_2_. The graphene nanoribbons suspension was prepared using NMP as solvent to obtain 0.5 mg mL^−1^. The mixture was treated for 2 min using an ultrasonic probe with amplitude of 70%. The GCE surface was modified using drop-casting technique by adding 10 µL of the GNR suspension. Then, modified electrodes were dried at 70 °C in an oven.

#### Preparation of the samples for AFM, SEM and Raman measurements

Samples for AFM measurements were prepared on silicon substrates due to their extreme flatness and availability. The reason for that choice is that graphene nanoribbon suspension contained different aggregates of GNRs with a relatively high scattering in size. Thus, in order to be able to characterize by AFM the smaller ones, a drop of 5 μL of GNRs exfoliated in NMP was deposited on the silicon surface and left to dry in an oven at 70 °C. In this way, with the aid of the optical microscope attached to the AFM microscope, it was possible to address the tip to those small spots where flat structures were observed and, therefore, avoid the larger and rougher agglomerates. It is worth noting that the high reflectance of silicon allowed to obtain the sufficient optical contrast to locate these small spots, which was not possible on the rougher and black, i.e. poor reflecting, glassy carbon. Samples for SEM and Raman measurements were prepared by depositing 5 μL of GNRs exfoliated in NMP on glassy carbon and gold surfaces, respectively, and left to dry in an oven at 70 °C.

#### Real sample preparation

Commercial EPI injection ampoules for parenteral administration (from B. Braun medical) were analyzed. Each 1 mL of clear, colorless, aqueous solution contains 1 mg of epinephrine bitartrate with sodium metabisulphite and sodium chloride as excipients. The sample was diluted with 0.1 M phosphate buffer solution (pH 8.0) and placed into the voltammetric cell to be analyzed without further pretreatment.

## Conclusions

A novel modified GCE was fabricated by simple incorporation of GNRs, previously synthesized by a chemical route, and applied to epinephrine determination. The presence of GNRs on the electrode surface increases both the charge electron transfer and the effective surface area of the electrode leading to an improved electrochemical response, in terms of higher current intensities and lower potential values, of redox compounds in solution. AFM images combined with Raman spectra show that structurally-integral GNRs form agglomerates by the stacking of flakes with lateral dimensions of several microns, thickness in the 8–34 nm range and surface roughness of ~ 0.4 nm. The analytical parameters found for our sensor are similar to those obtained for other sensors reported in the literature, but in our case the sensor construction is simpler and the potential of detection (E = −0.25 V) more adequate to avoid interferences. The applicability of the sensor for EPI determination in pharmaceutical samples was evaluated with satisfactory results. These results demonstrate that GNRs synthesized by a chemical route can be successfully applied for developing electrochemical sensors.

## Supplementary information

Supplementary Information.
