# Supplementary material for: Chemically synthesized chevron-like graphene nanoribbons for electrochemical sensors development: determination of epinephrine
Source: Sci Rep. 2020 Sep 3;10:14614. doi: 10.1038/s41598-020-71554-1 (PMC7471882; doi:10.1038/s41598-020-71554-1)
Supplement: Supplementary file 1 — Supplementary Information. [file 41598_2020_71554_MOESM1_ESM.docx]

Chemically synthesized chevron-like graphene nanoribbons for electrochemical sensors development: determination of epinephrine

Raquel Sainz^a^, María del Pozo^a^, Manuel Vilas-Varela^b^, Jesús Castro-Esteban^b^, María Pérez Corral^a^, Luis Vázquez^c^, Elías Blanco^a^, Diego Peña^b^, José A. Martín-Gago^c^, Gary J. Ellis^d^, María Dolores Petit-Domínguez^a^, Carmen Quintana^a^, Elena Casero^a^

*^a^Departamento de Química Analítica y Análisis Instrumental. Facultad de Ciencias. c/ Francisco Tomás y Valiente, Nº7. Campus de Excelencia de la Universidad Autónoma de Madrid. 28049 Madrid. Spain*

*^b^Centro Singular de Investigación en Química Biolóxica e Materiais Moleculares (CIQUS) and Departamento de Química Orgánica. Universidade de Santiago de Compostela. 15782 Santiago de Compostela. Spain.*

*^c^Materials Science Factory, Instituto de Ciencia de Materiales de Madrid (CSIC). c/ Sor Juana Inés de la Cruz Nº3. Campus de Excelencia de la Universidad Autónoma de Madrid. 28049 Madrid. Spain*

*^d^Departamento de Física de Polímeros, Elastómeros y Aplicaciones Energéticas,*

*Instituto de Ciencia y Tecnología de Polímeros (ICTP-CSIC), c/ Juan de la Cierva, 3, 28006 Madrid, Spain.*

* Corresponding author: [elena.casero@uam.es](mailto:elena.casero@uam.es)

**Figure S1**. A) Cyclic voltammograms of 1.0 mM Ru(NH_3_)_6_^2+/3+^ in 1M KCl (scan rate 10 mV s^-1^) at GCE (a) and GNRs/GCE (b), B) Plot of the cathodic and anodic peak currents vs. the square root of scan rate (v^1/2^) obtained from CVs registered at GCE (a) and GNRs/GCE (b) in the same solution.

**Figure S2**. Cyclic voltammograms of 1.0 mM EPI in phosphate buffer pH 8 at GNRs/GCE for different scan rates (10 - 300 mV·s^-1^). Inset: plot of cathodic peak current I_P3_ vs. the square root of scan rate (v^1/2^).

**Figure S3**. Cyclic voltammograms recorded with GNRs/GCE in aqueous buffer solution of 1.0 mM EPI at pH = 3.0 (solid line), pH = 6.0 (dashed line) and pH = 8.0 (dotted line).

**
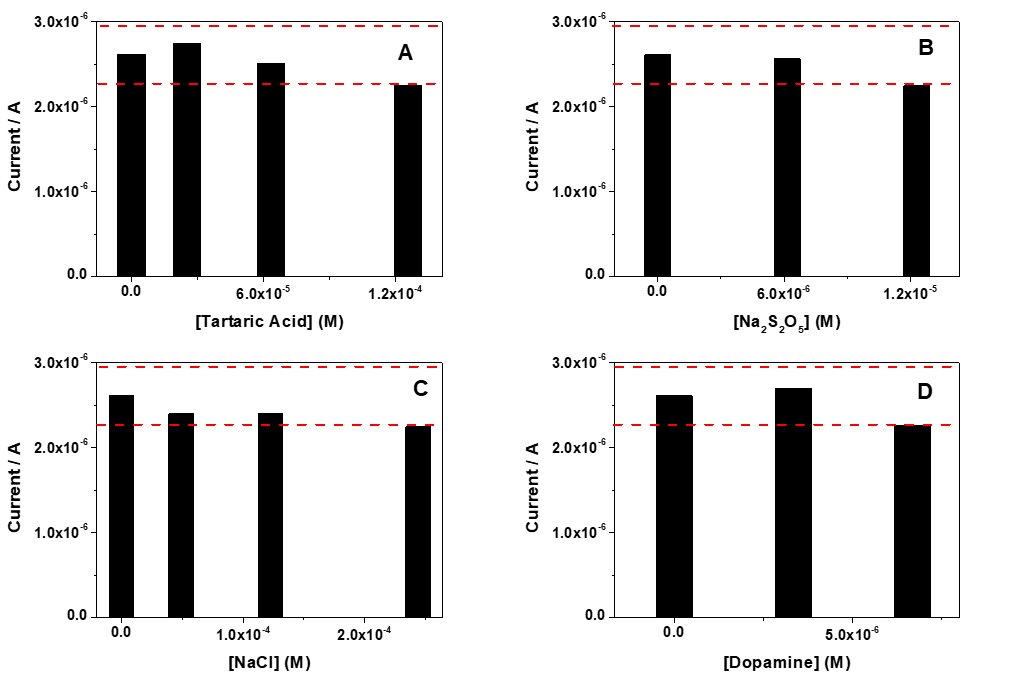
**

**Figure S4**. Interference study. Responses recorded with GNRs/GCE in 0.1 M phosphate buffer pH 8 containing 2.5 10^-5^ M EPI in the absence and in the presence of increasing amounts of A) tartaric acid, B) sodium metabisulphite, C) sodium chloride and D) dopamine. Red lines indicate variation on the initial EPI analytical signal equal to the maximum relative error (Er =13%).

**Figure S5**. Real sample determination by addition standard method: differential pulse voltammograms recorded with GNRs/GCE in a 166-fold diluted pharmaceutical sample in phosphate buffer pH 8 before and after successive addition of EPI. Inset: calibration plot.

| **E_0_ (V)** | **t (s)** | **E_i_ (V)** | **E_f_ (V)** | **a (mV)** | **v (mV s^-1^)** |
| --- | --- | --- | --- | --- | --- |
| 0.6 | 60 | 0.6 | -0.9 | 90 | 30 |

**Table S1**. DPV parameters optimized for EPI determination

| **Electrode** | **Technique** | **Linear range (µM)** | **Detection limit (µM)** | **Reference** |
| --- | --- | --- | --- | --- |
| MC-CNPE | DPV | 0.09 – 750.0 | 0.049 | ^54^ |
| PPy/AuNPs/SWCNTs-AuE | DPV | 0.004 – 0.1 | 0.002 | ^55^ |
| NP Au film | DPV | 20 – 190 | 2.43 | ^56^ |
| GCE/MWCNT/CA | DPV | 60 – 170 | 10 | ^57^ |
| IL/CNTPE | DPV | 0.3 – 450 | 0.09 | ^58^ |
| PXSP/GCE | DPV | 2 – 390 | 0.1 | ^59^ |
| SPE/CB-ERGO | SWV | 9.9 – 95 | 1.8 | ^60^ |
| poly(taurine)/GCE | DPV | 2 – 600 | 0.3 | ^61^ |
| PSRA/ modified CNTPE | CV | 2 – 64 | 0.1 | ^62^ |
| Poly (L-Asp)/ERGO/GCE | SWV | 0.1 – 110 | 0.025 | ^63^ |
| FePc/CPE | DPV | 1-30 | 0.5 | ^64^ |
| MWCNT/Fe_3_O_4_/2,3-Nc/GCE | DPV | 7.5-48 | 12.3 | ^65^ |
| MWCNT/Fe_3_O_4_/29H,31H-Pc/GCE | DPV | 7.5-48 | 4.6 | ^65^ |
| MWCNT/ZnO/2,3-Nc/GCE | DPV | 7.5-48 | 7.6 | ^65^ |
| MWCNT/ZnO/29H,31H-Pc/GCE | DPV | 7.5-48 | 6.5 | ^65^ |
| MWCNT-CH/GCE | DPV | 22.5-547 | 3.92 | ^66^ |
| Pt-NiO/MWCNTs/GCE | DPV | 0.5-300 | 0.035 | ^67^ |
| GNRs-GCE | DPV | 6.4 – 100 | 2.1 | This work |

MC: Molybdenum (VI) complex; CNPE: Carbon Nanotubes Paste Electrode; PPy: Polypyrrole; AuNPs: gold nanoparticles; SWCNTs: single-walled carbon nanotubes; AuE: gold electrode; NP: nanoporous thin; GCE: glassy carbon electrode; MWCNT: Multi-Walled Carbon Nanotubes; CA: Cellulose Acetate; IL: ionic liquid; CNTPE: Carbon Nanotubes Paste Electrode; PXSP: Poly(p-XylenolSulfonePhthalein); SPE: Screen Printed Electrodes; CB: Carbon Black; ERGO: Electrochemically Reduced Graphene Oxide; PSRA: Poly(Solid Red A);.FePc: Iron phtalocyanine; 2,3-Nc: naphtalocyanine; 29H,31H-Pc: phtalocyanine; CH:chitosan

**Table S2.** Comparison of the analytical response for different modified electrodes for EPI determination

| **EPI added (M)** | **EPI found (M)** | **Recovery (%)** |
| --- | --- | --- |
| 3.28 10^-5^ | 3.30 10^-5^ | 101 |
| 3.28 10^-5^ | 3.50 10^-5^ | 107 |
| 3.28 10^-5^ | 3.40 10^-5^ | 104 |

**Table S3.** Recoveries obtained from the analytical determination of EPI in a pharmaceutical product
